# Endovascular repair and open repair surgery of thoraco-abdominal aortic aneurysms cause drastically different types of spinal cord injury

**DOI:** 10.1038/s41598-021-87324-6

**Published:** 2021-04-09

**Authors:** Hamdy Awad, Esmerina Tili, Gerard Nuovo, Hesham Kelani, Mohamed Ehab Ramadan, Jim Williams, Katherine Binzel, Jayanth Rajan, David Mast, Alexander A. Efanov, Kareem B. Rasul, Sarah Moore, Michele Basso, Adel Mikhail, Mostafa Eltobgy, Raphael A. Malbrue, Eric Bourekas, Michael Oglesbee, Valerie Bergdall, Michael Knopp, Jean-Jacques Michaille, Hosam El-Sayed

**Affiliations:** 1 Department of Anesthesiology, Wexner Medical Center, College of Medicine, The Ohio State University, 410 W. 10th Ave, Columbus, OH 43210 USA; 2 Department of Cancer Biology and Genetics, College of Medicine, Wexner Medical Center, The Ohio State University, 460 W 10th Ave, Columbus, OH 43210 USA; 3 Phylogeny, 1476 Manning Pkwy, Powell, OH 43065 USA; 4 New York-Presbyterian/Weill Cornell, New York, NY 10021 USA; 5 Department of Radiology, Wexner Medical Center, College of Medicine, The Ohio State University, 410 W. 10th Ave, Columbus, OH 43210 USA; 6 ECMO Coordinator, Wexner Medical Center, The Ohio State University, Columbus, OH 43210 USA; 7 Department of Anesthesiology and Perioperative Medicine, University Hospitals, Cleveland, OH 44106 USA; 8 Department of Veterinary Clinical Sciences, The Ohio State University, 601 Vernon Tharp St., Columbus, OH 43210 USA; 9 School of Health and Rehabilitation Sciences, The Ohio State University, 106A Atwell Hall, 453 W. 10th Ave., Columbus, OH 43210 USA; 10 Department of Veterinary Preventive Medicine, University Laboratory Animal Resources, The Ohio State University, 111 Wiseman Hall, 400 West 12th Avenue, Columbus, OH 43210 USA; 11 Department of Veterinary Biosciences, The Ohio State University, 205 Goss Laboratory, 1925 Coffey Rd, Columbus, OH 43210 USA; 12 BioPerox-IL, Faculté des Sciences Gabriel, Université de Bourgogne-Franche Comté, 6 Bd. Gabriel, 21000 Dijon, France; 13 Department of Surgery, Division of Vascular and Endovascular Surgery, Eastern Virginia Medical School, 600 Gresham Dr, Norfolk, VA 23507 USA

**Keywords:** Preclinical research, Diseases of the nervous system, White matter injury

## Abstract

Both endovascular repair (EVR) and open repair (OR) surgery of thoraco-abdominal aortic aneurysms cause spinal cord (SC) injury that can lead to paraparesis or paraplegia. It has been assumed that mechanisms responsible for SC damage after EVR are similar to those after OR. This pilot study compared the pathophysiology of SC injury after EVR versus OR using a newly developed EVR dog model. An increasing number of stents similar to those used in patients were inserted in the aorta of three dogs to ensure thoracic or thoracic plus lumbar coverage. The aorta of OR dogs was cross-clamped for 45 min. Behavior assessment demonstrated unique patterns of proprioceptive ataxia and evolving paraparesis in EVR versus irreversible paraplegia in OR. MRI showed posterior signal in lumbar SC after EVR versus central cord edema after OR. Histopathology showed white matter edema in L3–L5 localized to the dorsal column medial lemniscus area associated with loss of myelin basic protein but not neurons after EVR, versus massive neuronal loss in the gray matter in L3–L5 after OR. Metabolome analysis demonstrates a distinctive chemical fingerprint of cellular processes in both interventions. Our results call for the development of new therapeutics tailored to these distinct pathophysiologic findings.

## Introduction

The number of patients with thoraco-abdominal aortic aneurysms continues to rise, with around 15,000 deaths per year in the USA resulting from thoraco-abdominal aortic aneurysm rupture. Surgical repair of thoraco-abdominal aortic aneurysms has led to a substantial decrease in the thoraco-abdominal aortic aneurysm mortality rate, given that the rupture is fatal in greater than 90% of cases. The correction can be done via open repair (OR) surgery or, more recently, through endovascular repair (EVR). Like OR, however, EVR carries significant risk of paraparesis or paraplegia. Thus, the incidence of SC injury associated with paraparesis or paraplegia following EVR surgery varies from 0.26 to 20%, depending on the nature, location and extent of the aortic aneurism, the presence or absence of comorbidity, the extent and location of stent coverage, and the strategy used for data collection^1–10^.

We have previously established a dog model of OR where transient cross-clamping of aorta results in gray matter damage and paraplegia^11^. We have also developed a mouse model of transient aortic cross-clamping confirming that paraplegia is due to massive gray matter damage^12,13^. We have shown that ischemia/reperfusion caused by transient aortic cross-clamping initiates a series of secondary events involving blood-spinal cord (SC) barrier damage, followed by vascular leakage and the development of central cord edema that ultimately results in gray matter damage and neuronal death^13^. Previous attempts to develop a model of EVR by ligating the segmental arteries in either small^14^ or large^15,16^ animals, or even a previous attempt at aortic stenting in sheep^17^ have focused exclusively at the thoracic level. As stenting is not possible in small animals, and there is very limited information on the pathophysiology of SC damage after EVR due to the lack of available SC tissue from patients and the absence of a preclinical vertebrate model, we initiated a pilot study on three male dogs whose thoracic and, for two of them, lumbar aorta was permanently covered by stents identical to those used for patients. The purpose of this work is to detail the pathophysiology of SC injury following EVR versus the pathophysiology after OR in our two dog models.

## Methods

### Animals

The Animal Care and Use Committee at the Ohio State University approved all the experiments with animals. This investigation conforms to the Guide for the Care and Use of Laboratory Animals published by the NIH. Three groups of male Greyhound dogs (around 25 kg, 15–21mo of age at the time of surgery) were randomly assigned to control (n = 4), OR (n = 4), and EVR (n = 3) groups. See Supplementary Table 1 for the workflow design.

### OR and EVR procedures

In the control group the surgery consisted of a left 4th space lateral thoracotomy and exposure of the aorta. In the OR the left thoracotomy, conducted like in control group, was followed by 45 min of aortic cross-clamping and permanent ligation of the top three paired thoracic segmental arteries^11^. In the EVR, left lower flank abdominal incision and retroperitoneal exposure of the distal abdominal aorta and bilateral iliac arteries was followed first by ligation of bilateral internal iliac arteries and the distal (7th) pair of lumbar segmental arteries, consequently decreasing the blood supply to the lower abdominal region.

EVR dog#1 first received a 21mm × 10 cm CTAG endograft (W. L. Gore & Associates, Flagstaff, AZ) to cover the origin of the left subclavian artery with its proximal edge overlapping the distal part of the origin of the innominate artery. As this was difficult due to the anatomic conformation (bovine arch), the left subclavian artery of EVR dog#2 and dog#3 was instead plugged using a 12-mm Amplatzer-II device (St Jude Medical, Inc, St Paul, MN). We then introduced another stent of the same size to cover the left subclavian origin all the way to the distal edge of the innominate artery. A third stent of the same size was used to extend the coverage to just proximal to the origin of the celiac artery. In addition, the lumbar segmental arteries (excluding the first and second pairs) of EVR dog#2 and dog#3 were covered using two VIABHAN 10mm × 5 cm stents (W. L. Gore & Associates, Flagstaff, AZ). As a consequence, segmental arteries were completely occluded at both the T1–T13 thoracic and L3–L7 lumbar levels in dog#3, while the 5th lumbar segmental arteries were left open in dog#2. Of note, the unpaired celiac and cranial mesenteric arteries, as well as the paired renal arteries, were left patent in the three dogs (see Supplementary Methods). Anesthetics and analgesics agents administered before, during and after surgery are presented in Supplementary Table 2. For euthanasia, animals maintained under anesthesia were intravenously injected with Euthasol (390 mg/ml) at 1 ml/ 10 pounds body weight at day 2 (control and OR) and day 9 (EVR) by an anesthesiologist and a veterinary specialist.

### Behavior assessment

Behavioral assessment, conducted by a veterinary specialist of dog behavior, aimed to qualitatively compare the initial effects of EVR versus OR in the SC in this pilot study. It included a complete neurologic examination (e.g. mentation, gait, cranial nerves, segmental spinal reflexes, muscle tone, paraspinal hyperesthesia) and descriptive open field locomotor assessment. All assessments were performed at baseline, 12 h after surgery, and daily until termination of the study.

### Computed tomography angiogram (CTA) and magnetic resonance imaging (MRI)

Animals had CTA and MRI of the SC at the Wright Center of Innovation in Biomedical Imaging at The Ohio State University to verify the occlusion of arteries and to analyze edema and localize the SC damage due to each intervention, respectively. Animals were handled blindly in both cases.

A multiphasic CTA was obtained from the neck to the pelvis of all dogs before the procedure, 48 hours after the procedure, and 9 days later for EVR dogs, while animals were under anesthesia. The CTAs were performed on a Philips 64 slice Brilliance CT scanner, 120 kV with 300 mAs, a 1.5mm slice thickness, pitch was 0.891 and 64×0.625 collimation, reconstructed with a 512×512 matrix. A pre-contrast whole body scan, a post-contrast angiographic phase CTA, a venous phase and delayed post-contrast images of the whole body 4–5 min post contrast injection were obtained.

MRI was performed on a Philips Ingenia CX 3Tscanner at the same time as CTA. MRI imaging included: T2-weighted images in all 3 planes (sagittal, coronal, and axial orientations), T1-weighted Turbo Spin-Echo (TSE) pre- and post-contrast in all 3 planes, and a T1-weighted SPIR (Spectral Presaturation with Inversion Recovery) pre- and post-dynamic contrast enhanced (DCE) images in the sagittal plane. A highly T2W fat suppression sequence was done to exclusively visualize the lumbar SC. Fat suppression T1W was run prior to the contrast administration. DW-MRI (DWI) and Diffusion Tensor Imaging (DTI, 6 directions) were performed with echo planar imaging (EPI) single-shot sequences. A 3D fast field echo (FFE) sequence was used to perform DCE-MRI with the IV injection of a Gd-based contrast agent.

### X-rays

Access into the distal abdominal aorta just above the external iliac origins was performed using micropuncture needle and wire, followed by micropuncture sheath. A stiff Glidewire was then inserted through the sheath under fluoroscopy using OEC 9800 C-Arm. A 5-Frensh short sheath was used to replace the micropuncture sheath. The Glidewire was passed all the way to the ascending aorta under fluoroscopy. A 5-F 65 cm Bernstein catheter was then passed over the wire to the ascending aorta. A 180 cm curved Lunderquest stiff wire was passed through the catheter to replace the Glidewire and having its curved end against the aortic valve. The dogs were fully heparinized at this point. The catheter and sheath were removed and replaced by a large sheath in place (In the first dog a 20-F Cook sheath was used, in the second and third dogs an 18-F Dryseal Gore sheath were used). The large sheath was hubbed with the tip in the mid-descending thoracic aorta. The Glidewire was then placed in a coaxial fashion all the way to the arch of the aorta followed by the Bernstein catheter and an arch angiogram was performed to identify the origin of the great arch vessels in a left anterior oblique view while the stiff wire was in place to avoid changing the arch anatomy with the stiff wire if the angiogram was performed without the stiff wire in place. The innominate artery and left subclavian arteries were marked on the flouro screen and the C-arm was locked in position.

### Tissue preparation and immunohistochemistry, quantification, and co-expression analyses

The SCs were removed en bloc and placed in ice within 30 minutes of sacrifice. One to two cm sections of each cord segment from C1–C7, T1–T13, L1–L7 and S1–S3 were carefully dissected from the en bloc resection. About half of the tissue from C1, C3, C5, C7, T1, T3, T5, T7, T9, T11, T13, L1, L3, L5, L7, S1, and S3 segments were placed in 10% buffered formalin and fixed for 48 hours, then embedded in paraffin. The remainder of the tissue was stored at − 80 °C for metabolome analyses. All sections were subsequently analyzed blindly.

### Immunohistochemistry (IHC)

All sections were dealt with blindly using an automated Leica Bond Max platform at Phylogeny Inc. (Powell, OH). All immunohistochemistry images were obtained using a ZEISS AXIO Imager light field microscope. Magnifications included 200x, 400x, and 1000x. The antibodies used for IHC were from: Abclonal: Gasdermin D (A10164); Enzo Life Sciences: S100 (ALX-810-220-R500); Parp3 (ALX-210-971-R100); or Abcam: CD31 (ab28364); GFAP (ab7260); Iba1 (ab5076); Myelin basic protein (ab209328); NeuN (ab177487); Neurofilament heavy polypeptide (ab7795); Pyruvate dehydrogenase (ab180759); and TMEM119 (ab185333). The intensity readings and co-expression analyses were done using the computer-based Nuance software (Perkin Elmer). The Nuance software allows for co-expression analyses of two targets by separating the two-color based images, converting them to a fluorescent type image, then merging the images while determining the percentage of cells that contain the two targets of interest. The Nuance software also includes the Inform System, which quantifies the number of cells of a given cell type that contain the target protein of interest. Quantification of a given protein was done using the computer-based InForm system (Perkin Elmer) in which the relative expression of a given protein is determined for a population of cells in a given tissue. Co-expression analyses were done using the Nuance system (CRI). In brief, a given tissue was tested for two different targets using fast red, NBT/BCIP or DAB as the chromogens. The results were then analyzed by the Nuance and InForm systems with a Zeiss Axioskop microscope to determine what percentage of cells were co-expressing the two targets of interest.

### Metabolome analysis

Samples from the SC of the 4 control, 4 OR and 3 EVR dogs were analyzed at Metabolon, Inc. (Morrisville, NC, USA) using an HD4 platform, that identifies different types of macromolecules, and a CLP platform aimed at specifically studying lipid alterations. Both platforms utilize Ultrahigh Performance Liquid Chromatography-Tandem Mass Spectroscopy (UPLC-MS/MS). Samples were processed as previously described^18^ (see Supplementary Methods for details). Welch’s two-sample *t*-test was used to test whether two unknown means were different from two independent populations. Matched pairs *t*-test was used to test whether two unknown means were different from paired observations taken on the same subjects. *P*-values were considered significant when ≤ 0.05. False Discovery Rate (FDR) for the sets of compounds were estimated using the *q*-values. Principal components analysis, an unsupervised analysis that reduces the dimension of the data, was use to emphasize the differences in metabolite changes between the EVR and OR groups. Each principal component is a linear combination of every metabolite and the principal components are uncorrelated.

## Results

### Development of an EVR dog model with increasing number of occluded segmental arteries

**Figure 1:**
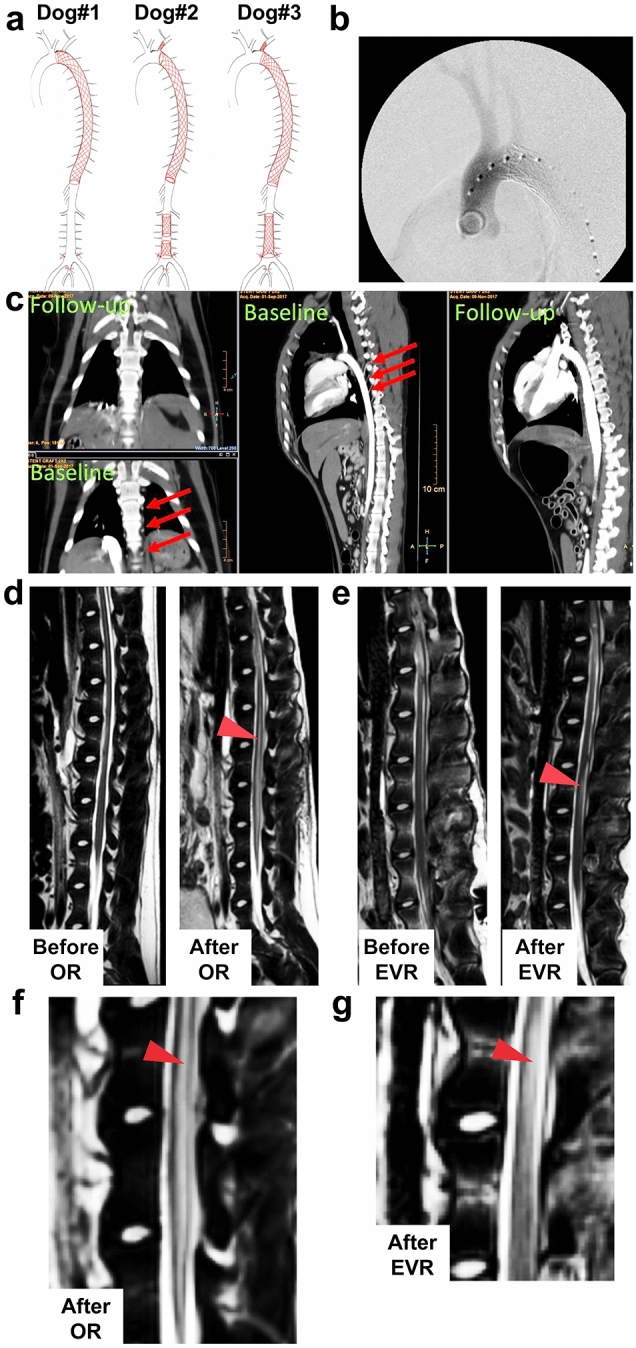
EVR and OR give different signals in MRI. (**a**) Drawing showing the extent of arterial occlusion in EVR dogs #1–3 as indicated. (**b**) X-ray images showing the stent inside the aorta of dog#1. (**c**) CTA image showing frontal (two left panels) and lateral (two right panels) views of EVR dog#3 before (baseline) and after (day 2) occlusion following EVR. Arrows point at segmental arteries before aortic stenting. (**d**) Sagittal T2-weighted MRI images: left, before OR surgery, showing no signal; right, after OR, showing abnormal signal centrally within the cord involving grey matter (arrowhead). (**e**) Sagittal T2-weighted MRI images of EVR dog#3: left, before EVR surgery, showing no signal; right, after EVR, showing abnormal signal within the posterior aspect of the cord at L3 involving white matter (arrowhead). (**f**), (**g**) Enlargements from right panels in (**d**) and (**e**), respectively.

OR was conducted as previously described^11^. For EVR, we escalated the level of SC ischemic insult via increasing the number of occluded segmental arteries in the three stented animals (Fig. 1a–c). While only the descending thoracic artery was stented in dog#1, dogs #2 and #3 also received lumbar stents. Of note, the 5th pair of lumbar segmental arteries of dog#2 (the most frequent site of implantation of the great radicular artery, a.k.a. the Adamkiewicz artery^19^) was left patent between the two covering stents, but not in dog#3 where the stents were overlapped (Fig. 1a). As a consequence, segmental arteries were completely occluded at both the T1–T13 thoracic and L3–L7 lumbar levels in dog#3 (Fig. 1a, c).

### Neurologic and MRI correlates of EVR versus OR animals

Qualitative behavior assessment demonstrated unique patterns of SC injury between EVR and OR. The OR dogs showed spasticity and severe paraplegia without recovery, an irreversible complication that has been shown to be caused by massive gray damage in both dogs and mice^11–13^, and were thus euthanized on day 2. EVR dog#1 showed mild, transient asymmetric weakness and spasticity of the hind limbs but maintained the ability to ambulate. Motor deficits were no longer apparent by day 7, but spasticity persisted. Dog#2 developed severe, asymmetrical weakness of all four limbs with spasticity and inability to ambulate. Ambulation on three legs, with persistent motor deficits and ataxia, was recovered by day 6 with the left thoracic limb persistently unable to support weight. Proprioceptive placing (ability to right to paw from a knuckled position) was absent in the left thoracic and left pelvic limbs and was present but slow (>2 seconds) in the right thoracic and right pelvic limbs. Finally, dog#3 developed an initially profound asymmetrical non-ambulatory paraparesis but recovered ambulation by day 4. From day 2 to day 4, there was a severe proprioceptive ataxia in both pelvic limbs, but not in the thoracic limbs. From there, however, moderate proprioceptive ataxia and motor deficits were static and persisted until day 9. Of note, the conscious proprioception, i.e., the position sense as perceived at the cerebral level, is conveyed to the cerebrum primarily via the dorsal column / medial lemniscus pathways of the SC white matter^20^. Of note, the three EVR dogs had normal nociception, indicating that the axons localized in the anterolateral tracts (lateral funiculus) of the SC white matter remained functional. The MRI images corroborated the clinical findings and indicated that OR and EVR affected different areas of the SC when causing injury. OR dogs presented with an abnormal signal centrally within the cord involving edema and gray matter damage (Fig. 1d, f), as previously reported^13^. In sharp contrast, while EVR dogs #1 and #2 did not present any abnormal signal at day 2 and day 9, EVR dog#3 showed abnormal signal within the posterior aspect of the cord at L3 involving white matter at day 9 only (Fig. 1e, g). As the posterior aspect of the SC contains the ascending tracts transmitting proprioceptive sensations, this was in agreement with the behavior analysis. Given the above results, we then focused on comparing EVR dog #3 with EVR dog #1, as well as with OR and control dogs.

### Hematoxylin and eosin (H&E) stain analysis of SC lesions in EVR versus OR models

**Figure 2:**
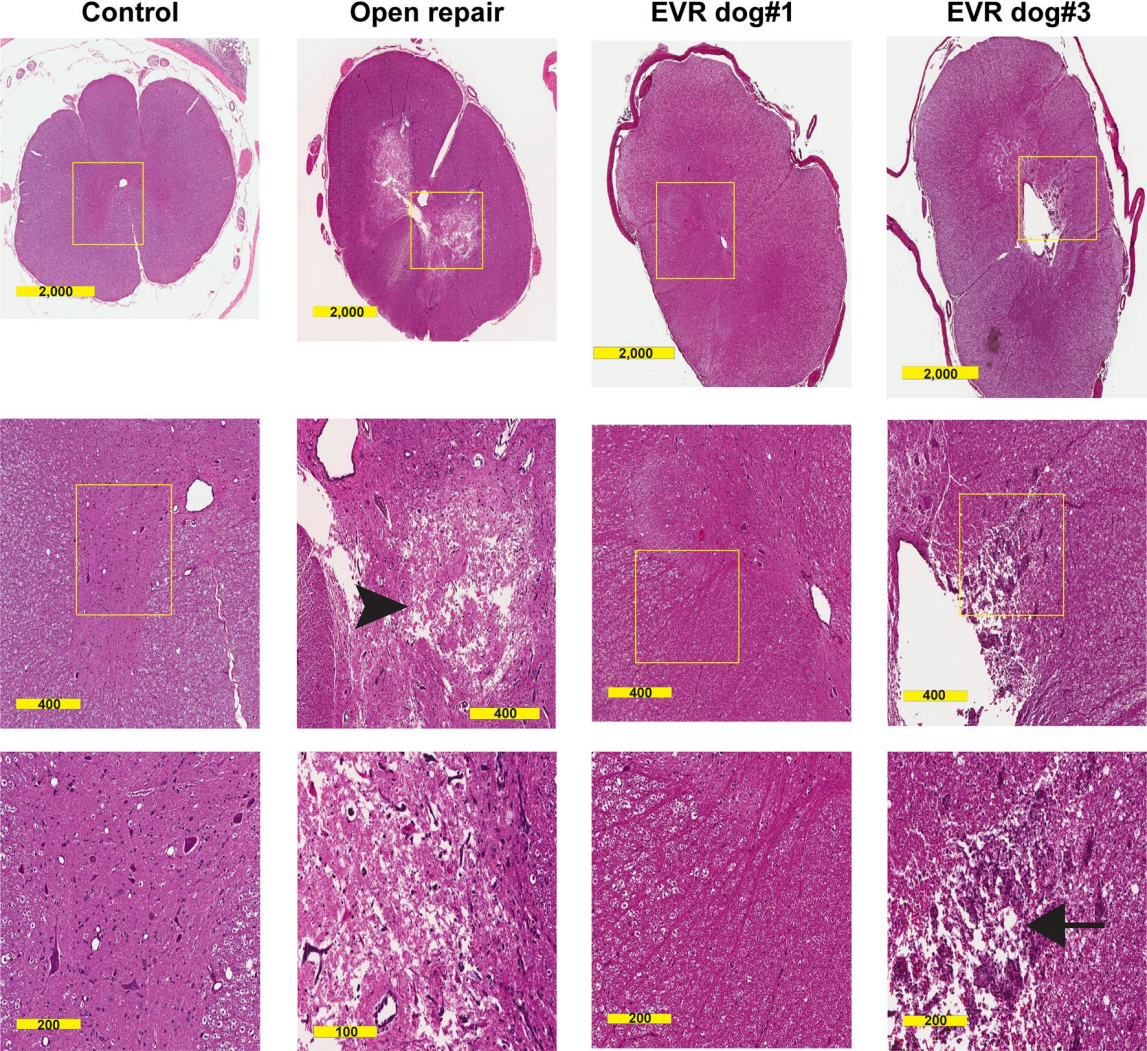
EVR and OR cause different types of SC injury. H&E staining of cross-sections of the SC from control, OR, and EVR dog#1 and dog#3 as indicated. Images from the second column from the left show extensive damage to anterior and lateral horns of the gray matter following OR surgery (arrowhead). In contrast, aortic stenting (EVR dog#1 and dog#3) primarily leads to white matter damage (two right columns). White matter damage was especially exacerbated in EVR dog#3 (arrow, with the yellow box corresponding to part of the posterior white column). Cross-sections were at L5 level. Scale bars are in µm.

H&E sections from each odd number SC segment from C1 to S3 were scored blindly for the following variables: edema, neuronal degeneration and/or loss, gliosis, endothelial cell degeneration, inflammatory cell infiltrates, pallor of white matter, pyroptosis, and apoptosis. After “unblinding”, analysis revealed marked differences between the OR and EVR models whereas no pathologic changes were evident in the controls. In accordance with our previous results^11–13^, the H&E findings in the OR where restricted to L3 and L5 where marked gray matter edema was present in both the ventral and dorsal horns and spared a thin lateral rim of the gray matter where it interfaced with the white matter. In the EVR, at the L5 level (a region covered by stents in dog#3 but neither in dog#1 and dog#2), the most striking histological changes were found in dog#3 that presented with marked damage to the posterior column of the white matter, directly adjacent to the posterior aspect of the dilated canal (Fig. 2). This result was in accordance with both the behavior and MRI analyses. In addition, the lateral and anterior white matter at L5 in dog#3 showed a pallor indicative of edema, a feature also present, although much less pronounced, in dog#1. Of note, this feature was not found in OR animals (Fig. 2). In addition, there was also evidence of white matter edema associated with peri-neuronal edema in the gray matter in the SC of stented dogs #1 and #3 at T1 level (not shown), in accordance with the fact that the segmental arteries in this region were occluded by stents in both animals. Finally, at L1 level, the appearance of white matter was close to normal in dog#1, while pronounced white matter edema was observed in dog#3 (not shown). Altogether, H&E analysis indicates that SC injury in the EVR model is primarily associated with white matter damage. While lumbar stenting caused some neuronal loss at the lumbar level in stented dog#3, massive neuron loss was observed at L5 in OR animals only.

### Immunohistochemical analysis of white matter and grey matter of the SC

**Figure 3:**
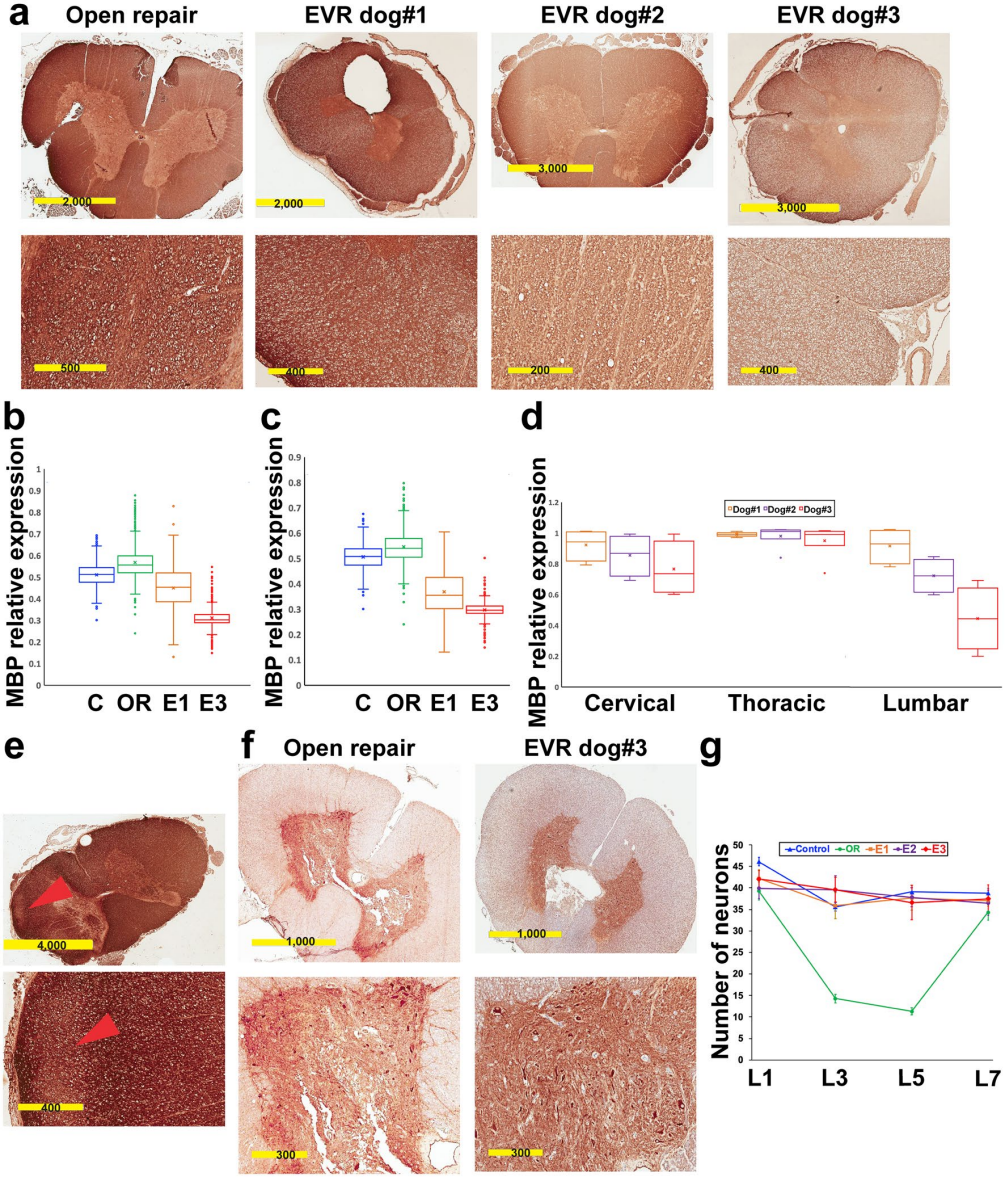
EVR primarily causes injury to the white matter. (**a**) Immunohistochemistry for myelin basic protein (MBP) in EVR dogs #1–3 versus OR. Cross-sections were at L5 level. (**b**), (**c**) Box and whisker plots showing MBP levels assessed by IHC on cross-sections of the SC throughout the L1–L5 region (**b**) or the T13–L3 region (**c**). C, control; OR, open repair; E1 and E3, EVR dog#1 and dog#3, respectively. Horizontal bars show the lower (first quartile), the median (second quartile) and the upper (third quartile) quartiles from bottom to top. The cross represents the mean value. (**d**) Box and whisker plots showing the dispersion of the mean levels of MBP expression in the cervical (C1, C3, C5 and C7), thoracic (T3, T5, T7, T9, T11 and T13) and lumbar (L1, L3, L5 and L7) regions of the SC of EVR dogs #1–3. (**e**) Reduced MBP level (arrowheads) in the cervical SC of EVR dog#2. (**f**) IHC for neurofilament heavy polypeptide in EVR dog#3 versus OR. Cross sections were at L5 level. (**g**) Neuronal density at the L1, L3, L5 and L7 levels in a control dog, an OR dog, and EVR dog#1 (E1), dog#2 (E2) and dog#3 (E3) was quantified by assessing the number of neurons per 10X field (given as mean ± standard deviation) as determined by pyruvate dehydrogenase testing. Scale bars are in µm.

To further clarify and quantify these observations, IHC was done in a blinded fashion using a panel of biomarkers that can isolate the different cell types, as well as myelin basic protein (MBP), a major component of white matter, and neurofilament heavy polypeptide, a major component of axons. At L5 level, MBP level decreased progressively in the white matter of EVR dogs as compared with OR (Fig. 3a). MBP expression was further analyzed in each consecutive odd SC segment of EVR and OR dogs and compared with the controls. While this study remains semi-quantitative, it nevertheless showed that the relative level of MBP throughout the L1–L5 part of the SC was roughly equivalent in the control, OR and EVR dog#1, but showed a tendency to reduction in dog#3, given that the third quartile (upper quartile, 75% values limit) of dog#3 was below the first quartile (lower quartile, 25% values limit) of control, OR and EVR dog#1 (Fig. 3b). Similarly, the distribution of MBP levels throughout the T13–L3 part of the SC was lower for both EVR dog#1 and dog#3 than for control and OR dogs, the third quartile of dog#1 and dog#3 being far below the first quartile of control and OR dogs, and the third quartile of dog#3 being just above the first quartile of dog#1 (Fig. 3c). If one considers the distribution of the mean MBP levels over the whole SC of EVR dogs, they were similar over the cervical and thoracic regions, however they showed tendency to reduction in the lumbar region in correlation with the increasing extent of stenting, where the third quartile of dog#2 was just above the first quartile of dog#1, and the third quartile of dog#3 was just above the first quartile of dog#2 (Fig. 3d). In addition, although no histologic changes were evident in the cervical region, a tendency to MBP reduction, apparent as less colored regions, was seen in the SC of all EVR dogs in this region (Fig. 3e), indicating that thoracic stenting was also causing some degree of damage to the white matter. This suggests that reduced MBP expression might be indicative of latent white matter injury in the EVR model before any MRI changes become apparent.

**Figure 4:**
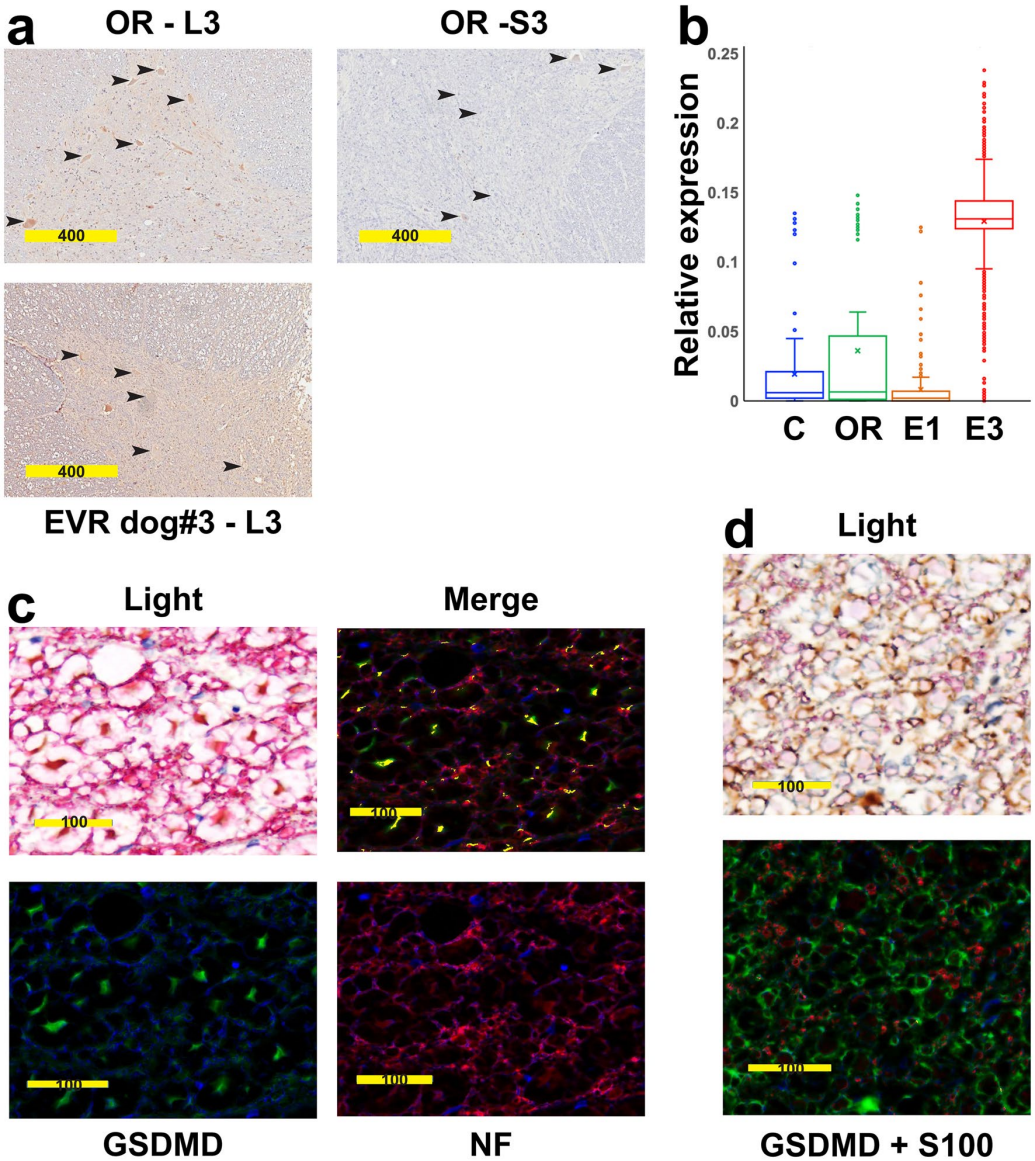
The SC of EVR and OR dogs express different markers of cell death. (**a**) IHC for Parp3 (brown) on cross-sections at L3 and S3 levels in an OR dog (top left and right panels, respectively), and at L3 in EVR dog#3. Arrowheads point at motor neurons. (**b**) Box and whisker plots showing Gasdermin D expression assessed by IHC on multiple lumbar cross-sections of a control dog (C), an OR dog, and EVR dog #1 (E1) and dog#3 (E3). The cross gives the mean value. (**c**) Bright field (upper left panel) and immunofluorescence (three other panels) showing co-localization between Gasdermin D (GSDMD, green) and neurofilament heavy polypeptide (NF, red) on L5 cross-sections from EVR dog#3. Yellow: co-expression. (**d**) Bright field (upper panel) and immunofluorescence (lower panel) showing lack of co-localization between Gasdermin D (GSDMD, green) and S100 (oligodendroglial marker, red) on L5 cross-sections from EVR dog#3. Scale bars are in µm.

In accordance with our previous results in mouse^13^, motor neurons at L3 but not at S3 in OR showed robust expression of PARP3, a factor implicated in apoptosis^21^. In contrast, PARP3 expression at L3 remained below the level of detection in the SC of EVR dog#3 (Fig. 4a). As Gasdermin D expression has been associated with white matter loss in multiple sclerosis as well as in experimental autoimmune encephalomyelitis^22,23^, we used a semi-quantitative analysis to assess Gasdermin D expression in our two dog models. The Gasdermin D level was elevated throughout the lumbar SC of EVR dog#3, but not in that of either EVR dog#1 or an OR dog (Fig. 4b). We further found in stented dog#3 that Gasdermin D at L5 level co-localized with the axonal marker neurofilament heavy polypeptide (Fig. 4c), but not with the oligodendroglial marker S100 (Fig. 4d). On the other hand, IHC for pyruvate dehydrogenase, a key component of viable neurons, confirmed the extensive damage to the gray matter at L5 level in OR dogs, versus the overall preservation of the gray matter in the SC of stented dog#3 (Fig. 3f). The number of neurons in the dorsal and ventral horns of the SC was similar in all cervical, thoracic, or sacral segments when comparing the controls to OR and EVR dogs (not shown). In contrast, there was a 60–70% decrease of the number of neurons in the L3 and L5 segments of the SC of OR dogs, but not the SC of EVR dogs (Fig. 3g). Of note, the L3 to L5 region corresponds to the lower enlargement of the SC that contains the motor neurons that innervate the muscles of the pelvic limbs. Although each SC segment was also tested for GFAP (astrocytes), S100 (oligodendrocytes), Iba1 and TMEM119 (microglial cells), and CD31 (endothelial cells) expression, we observed no difference in the number or distribution of the astrocytes, oligodendroglial or microglial cells with either the OR or EVR model versus the controls (data not shown). Altogether, histological analyses confirmed the qualitative differences between SC damage caused by EVR and OR in accordance with results obtained from behavior and MRI analyses.

### SC biochemical alterations in EVR versus OR

We then used metabolome analysis to identify biochemical changes associated with the pathophysiology of SC injury in OR versus EVR models. In the following analyses, the three EVR dogs were considered as a group, given that they all were submitted to aortic stenting, although at a variable extent. There were 511 named biochemicals in the HD4 dataset from these dog SC samples. At a statistical significance level of *p* < 0.05 (Welch’s two-sample *t*-test), 26 differences between groups can be expected from random chance. The OR (n = 4) to control (n = 4) comparisons (thoracic, 64; lumbar, 108) had the fewest significant differences, still well above the random chance level. The combined EVR group (n = 3) gave a large number of changes compared to either the control (thoracic, 350; lumbar, 360) or the OR group (thoracic, 288; lumbar, 309). In the CLP data set, 1002 lipids were detected, and an additional 15 class sum values were reported. At *p* < 0.05 (Welch’s two-sample *t*-test), 51 differences between groups can be expected from random chance. The OR to control comparison showed 111 (thoracic) and 22 (lumbar) differences, suggesting some level or no difference, respectively. In contrast, the EVR group had greater numbers of differences with both the control (thoracic, 310; lumbar, 138) and the OR group (thoracic, 73; lumbar, 188).

**Figure 5:**
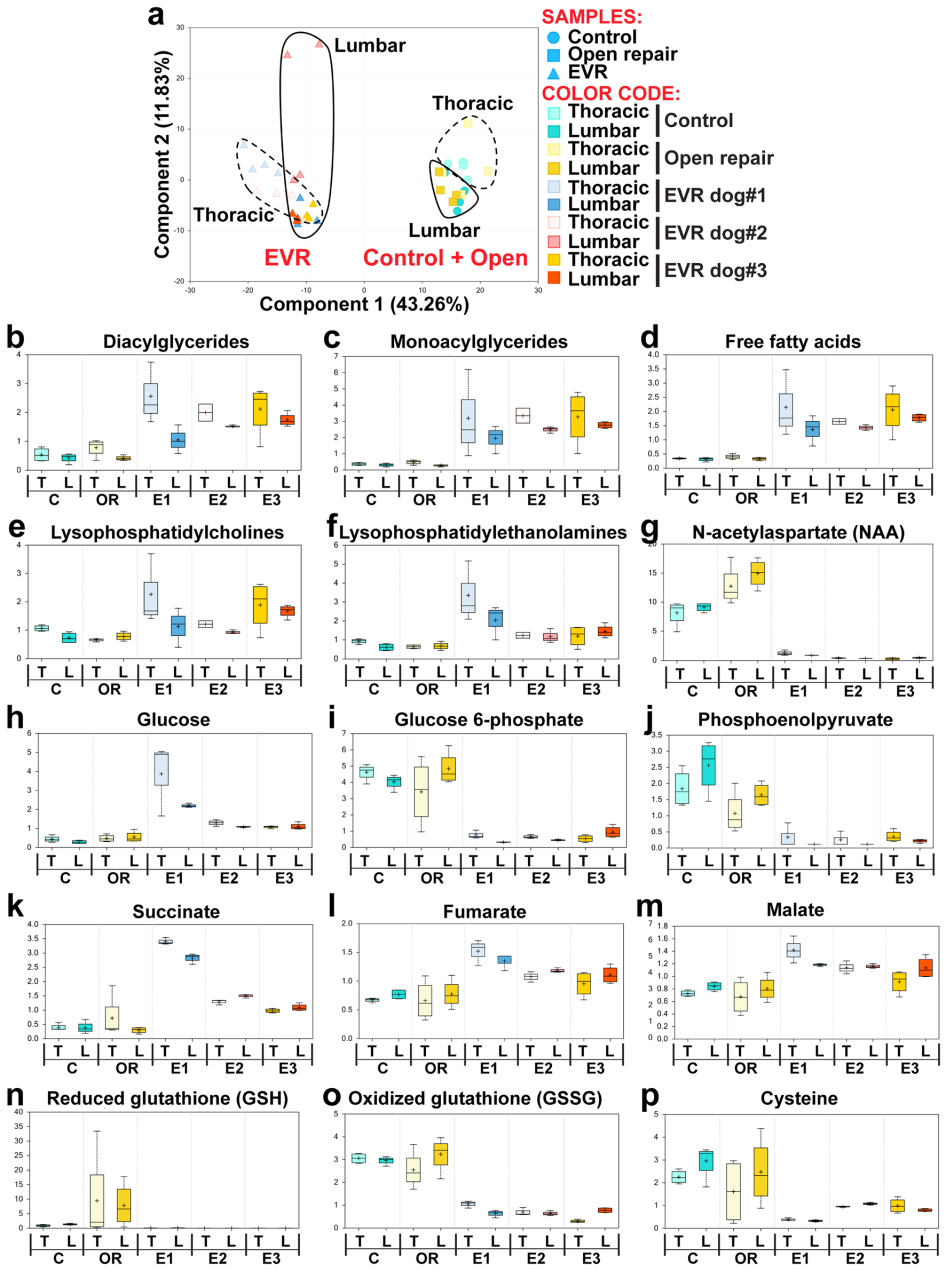
The SC of EVR and OR dogs show strikingly different types of biochemical changes. (**a**) Principal component analysis showing a clear separation between the samples from EVR dogs #1–3 and the clustered samples from control and OR dogs. There is also evidence of a more limited separation between the samples from thoracic and lumbar spinal cord. (**b**)–(**p**) Box and whisker plots showing the scale intensity for representative metabolites in the thoracic (T) and lumbar (L) SC of control dogs (n = 4), OR dogs (n = 4) and EVR dogs #1–3 (E1, E2 and E3, respectively). Results are presented separately for the three EVR dogs to illustrate the consistency of data between them, irrespective of the extent of stenting. Statistical analyses, however, were conducted using the three EVR dogs as a group. Significance was assessed using a Welch’s two-sample *t*-Test (*p* < 0.05).

A principal component analysis from the HD4 metabolome data (Fig. 5a) showed a clear separation between the biochemical changes in the three EVR dogs (n = 3), on the first hand, and both the control (n = 4) and OR (n = 4) data, on the second hand (43% of the variation for all changed biochemicals being explained by the type of surgery). There was less absolute separation between data from thoracic and lumbar SC (Fig. 5a), which was further confirmed by hierarchical clustering analyses (not shown). This was possibly due to the fact that both the thoracic and lumbar aorta were stented in dog#3 (see orange and dark orange triangles). A similar, although less distinct, separation was observed with the data from complex lipid panel metabolomic analysis (not shown). It should be noted, however, that the time difference at euthanasia between these two sets of samples cannot be fully discounted, i.e., even if the pathophysiological changes are most probably the primary driver of metabolomic differences, in agreement with the above results, there is still a difference in the time point post-surgery for these two major clusters.

Among the observed biochemical changes (all at *p* < 0.05), the EVR group showed elevated levels of diacylglycerides, monoacylglycerides, and free fatty acids (Fig. 5b–d), as well as an increase in lysophospholipids such as lysophosphatidylcholines and lysophosphatidylethanolamines (Fig. 5e, f). These metabolite changes are indicative of overall lipid breakdown in the EVR model, consistent with white matter injury and myelin loss. EVR dogs further showed dramatically reduced levels of N-acetylaspartate (NAA) (Fig. 5g), one of the most abundant brain metabolites that is found to be reduced in a majority of neuropathologies, including traumatic brain injury^24^. EVR samples in addition showed glucose levels higher than both control and OR samples, but reduced levels of glucose 6-phosphate and phosphoenolpyruvate (Fig. 5h–j). This is an indication of aerobic glycolysis being impaired due to hypoxia. In contrast, elevated levels of lactate (not shown) suggested a metabolic adaptation to hypoxia, given that glycolysis can function in hypoxic or anaerobic conditions. This was associated with elevated levels of succinate, fumarate, and malate, indicative of malfunctions in the Krebs cycle (Fig. 5k–m). Finally, the EVR group also showed highly reduced levels of both reduced glutathione (GSH) and oxidized glutathione (GSSG) (Fig. 5n, o), reduced levels of cysteine, the rate-limiting precursor for GSH biosynthesis^25^ (Fig. 5p), and reduced levels of cysteine-glutathione disulfide and S-methylglutathione (not shown), suggesting high levels of oxidative stress in the SC of EVR dogs. Altogether, the results from the metabolome analysis provide a strong evidence that the SC damage caused by EVR and OR surgeries is due to different pathophysiological mechanisms.

## Discussion

This pilot study reports the development of a large canine model of EVR using aortic stenting that results in transient paraparesis. Importantly, this is a model that rests on using the same stents and procedure used in humans, while nearly all other attempts to simulate EVR-induced paraparesis or paraplegia were conducted by permanently ligating a variable number of segmental arteries, usually at the thoracic level only^14–16^, with one experiment reporting thoracic stenting in sheep causing nearly no histological or behavior change over 7 days^17^, in agreement with our study. Ligated segmental arteries lack the dynamic nature of the changes in collateral blood flow that is seen after aortic stenting. Based on behavior, MRI, histopathological and metabolome data, the comparative analysis of our EVR model versus our previously developed OR model based on aortic cross-clamping^11^ demonstrated that the pathophysiology of EVR, which at the lumbar level exhibited primarily white matter damage with overall sparing of the gray matter at day 9 after surgery, is dramatically different from that of the OR, which showed massive gray matter damage as early as day 2, with no obvious white matter damage.

Stenting the entire thoracic region in human patients can be sufficient to induce paraparesis or paraplegia. In an attempt to simulate this clinical scenario, our EVR dog#1 had complete thoracic stent coverage in addition to obturation of the left subclavian artery and ligation of bilateral internal iliac arteries and the distal pair of lumbar arteries; however, despite this intervention, dog#1 only exhibited mild behavioral impairment, an outcome reminiscent of that previously experienced by other investigators after ligating some or all the thoracic segmental arteries in sheep or dog, or stenting the thoracic aorta in sheep^14–17^. This finding was likely due to several factors: (1) The tip of the dog SC reaches the 6th lumbar vertebra (L6)^20^ versus L1/L2 in human; (2) The SC lower enlargement, which harbors motor neurons that control the pelvic limbs, is most often located between L3 and L5 lumbar vertebrae in the dog versus T9–T11 thoracic vertebrae in human; (3) The artery of Adamkiewicz (a.k.a. the great radicular artery), that provides the major blood supply to the SC lower enlargement, originates on the left side of the aorta between the T8 and L1 vertebrae in 89% of people^26^, however, it usually originates between L4 and L6 vertebrae in dog most often at L5^19^; and (4) It has been found that damage or occlusion of the artery of Adamkiewicz can result in anterior spinal artery syndrome that impairs motor function of the posterior limbs^27^. The importance of the blood supply from the artery of Adamkiewicz is further emphasized by a study in Rhesus monkeys showing that ligating the anterior spinal artery above its junction with the artery of Adamkiewicz usually leaves the animal neurologically intact, while ligating the anterior spinal artery below this junction usually causes paraplegia^28^.

A major histologic correlate was edema in the posterior columns of the SC lumbar region. This was strongly associated with central canal enlargement, suggesting that blockage of blood flow to and from the region led to a regional increase in cerebrospinal fluid pressure. While the histology showed overall preservation of the SC tissue after EVR at day 9, a key finding was the dramatic loss of MBP expression around the nerve processes of the white matter. This loss was evident not only in the lumbar segments, where the edema was present, but also in cervical cord segments where no such edema was seen. Such a dramatic MBP reduction in the myelin would clearly cause malfunction in the associated neurons of the region. With regard to the molecular mechanisms, the IHC analysis showed loss of MBP expression and increased axonal Gasdermin D expression in EVR versus elevated PARP3 expression and neuronal loss in OR. Of note, PARP3 is also expressed in motor neurons of the SC in our OR mouse model (Awad et al., unpublished results). The fact that Gasdermin D has been implicated in secretion of proinflammatory IL-1β and IL-18 and induction of pyroptotic cell death^29^ suggests that the myelin changes in the SC of EVR animals are associated with high levels of inflammation at day 9 post-intervention, possibly due to the presence of fatty acids and other types of lipids. Whether this is indicative of imminent cell death will be investigated in future experiments.

The differences in histological and molecular findings between EVR and OR were reflected in the accompanying behavioral findings. Specifically, OR dogs exhibited irreversible spasticity and paraplegia after surgery, and this was associated with consistent behavioral, histologic, and MRI findings. The three EVR dogs, which experienced an escalating degree of aortic stenting coverage, showed an increasing amount of impairment. Dog#1 showed limited impairment, an outcome reminiscent of that previously experienced by other investigators after ligating some or all the thoracic segmental arteries in dog or sheep^15,16^ or stenting the thoracic aorta^17^. Dog#2 demonstrated moderate and reversible asymmetrical weakness of all four limbs associated with ataxia, and dog#3 exhibited initially profound paraparesis with ambulation recovery at day 4 but persistent moderate proprioceptive ataxia and motor deficits until day 9. Of note, the results of behavioral, histological and MRI analyses were consistent, as proprioceptive ataxia most likely resulted from the histological damage to the dorsal column of the lumbar SC white matter that translated into an abnormal MRI signal, while normal nociception was expected given the lack of histological damage at the level of the anterolateral system in the lumbar SC white matter.

The transient cross-clamping of the aorta in OR can induce reperfusion injury to the SC^11–13^, but the vascular insult in EVR is not associated with reperfusion injury. In EVR, the collateral vessels that directly serve the anterior spinal artery are permanently blocked and, therefore, the extent of remaining blood flow will determine the extent of SC damage. Increasing the extent of aortic stent coverage translated into MRI signals as well as a higher degree of neurologic symptoms and histological damage. Thus, our EVR dog model could also be used to study the mechanism of ischemic stroke due to complete obstruction of a major artery in the brain. It should be noted that, at the thoracic and lumbar levels, the SC is fed by blood primarily through the spinal arteries, although a network of interconnected capillaries runs at its surface. Specifically, there are two posterior spinal arteries that feed the posterior white matter of the SC, versus one anterior spinal artery that travels caudally down the SC through the anterior sulcus and primarily feeds the major part of the grey matter^30^. It is estimated that the anterior spinal artery, whose diameter is significantly larger than that of the two posterior spinal arteries, provides about 2/3 of the blood to the SC, versus about 1/3 for the two posterior spinal arteries. Thus, in a situation of hypoperfusion such as the one caused by stenting the thoraco-abdominal aorta, it can be expected that this difference in diameter translates into differences of hypoperfusion level (most of the remaining blood likely flows to the larger anterior spinal artery), resulting in starving the two posterior spinal arteries and the region they feed (the white matter of the dorsal column of the SC) from blood.

In addition, the metabolome analysis provided additional strong evidence that the pathophysiology of SC injury after EVR and OR are significantly different. In particular, the grouping of the three EVR dogs in our principal component metabolome analysis clearly displayed the common nature of the pathology caused by aortic stenting, irrespective of its extent. Lipidomics revealed increased levels of diacylglycerides, monoglycerides, free fatty acids, lysophosphatidylcholines and lysophosphatidylethanolamines in all EVR dogs, features previously found in pathologies associated with white matter damage in rat brain submitted to hypoxia or focal cerebral ischemia^25,31^. This is not surprising, for the lipid-rich SC is very dense in phospholipids, especially with respect to the myelin sheath. Lysophospholipids are derived from the lipase cleavage of an acyl-chain from phospholipids, and so these results fit well with white matter injury associated with myelin degradation. Also, it has been estimated that roughly 50% of the total energy expenses of the rat adult brain is delivered through fatty acid oxidation^32^, thus it is possible that fatty acids released from myelin degradation might be used as an additional source of energy in hypoxic conditions, provided that enough oxygen remains available. There were elevated levels of glucose associated with reduced levels of glucose-6-phosphate and phosphoenolpyruvate in the EVR model, which suggests a shift from aerobic to anaerobic glycolysis. Indeed, HIF-1 (Hypoxia-induced factor 1) activity allows cells to adapt to hypoxia by up-regulating anaerobic ATP production and down-regulating phosphorylative oxidations, in particular through increasing the rate of glycolysis and diverting fatty acids from catabolism to acetyl-CoA^33,34^. In addition, glucose produced from astrocytic glycogen can sustain neuronal activity in conditions of hypoxia, at least for some time, and glycolysis is considered a survival pathway^35^. Further, lactate and aspartate levels increased in the three EVR dogs (not shown), and these compounds can be used by astrocytes for gluconeogenic processes, with the advantage of limiting lactic acidosis^35^. Finally, glucose oxidized through the pentose-phosphate pathway serves to regenerate GSH^36^, which could also explain very low levels of oxidized glutathione (GSSG) along with undetectable levels of GSH.

In conclusion, we acknowledge that our pilot study has a few limitations, the first one being in the small number of dogs due to the prohibitive costs of stenting, and consequently the difficulty to reach significant statistical power analysis for quantitative parameters. On the other hand, the lack of aortic aneurisms, that can be of different origins (atherosclerosis caused by high blood pressure, unhealthy diet, or cigarette smoking, inflammatory diseases or genetic connective tissue disorders), allowed us direct, non-biased comparisons of the iatrogenic effects of both surgical interventions. Based on behavior, MRI, histopathological and metabolome comparisons, the results presented here already provide clear, strong evidence that the mechanisms of SC injury from EVR are drastically different from those from OR, and point to the necessity to develop specific therapeutical interventions. As the SC from patients is not available, future studies will concentrate on comparing the changes in nucleic acids, proteins and biochemicals in dogs and patients body fluids (cerebrospinal fluid, blood and urine), which should lead to designing new, innovative therapeutic interventions in the future.

## Supplementary Information

Supplementary Information 1.
