# Supplementary material for: Endovascular repair and open repair surgery of thoraco-abdominal aortic aneurysms cause drastically different types of spinal cord injury
Source: Sci Rep. 2021 Apr 9;11:7834. doi: 10.1038/s41598-021-87324-6 (PMC8035135; doi:10.1038/s41598-021-87324-6)
Supplement: Supplementary file 1 — Supplementary Information 1. [file 41598_2021_87324_MOESM1_ESM.pdf]

Endovascular repair and open repair surgery of thoraco-abdominal aortic aneurysms cause drastically different types of spinal cord injury

Hamdy Awad[1,*], Esmerina Tili[1,2], Gerard Nuovo[3], Hesham Kelani[1], Mohamed Ehab Ramadan[4], Jim Williams[3], Katherine Binzel[5], Rajan Jayanth[1], David Mast[6], Alexander A. Efanov[1], Kareem B. Rasul[7], Sarah Moore[8], D. Michele Basso[9], Adel Mikhail[3], Mostafa Eltobgy[1], Raphael A. Malbrue[10], Eric Bourekas[5], Michael Oglesbee[11], Valerie Bergdall[10], Michael Knopp[5], Jean-Jacques Michaille[2,12] & Hosam El-Sayed[13]

Supplementary Methods

Supplementary Table 1. Workflow of the study.

**Group**	**Number of dogs**	**Intervention**	**Imaging**	**Euthanasia**	**Metabolomics**
Control	4	Opening and closing of the chest as in OR.	MRI + CTA before surgery, and at day 2 (before euthanasia).	48 hours post-surgery.	-**Thoracic**: 4 dogs, 1 sample / dog.<br>- **Lumbar**: 4 dogs, 1 sample / dog.
Open repair (OR)	4	**Transient** (45 min) aortic cross-clamping.	MRI + CTA before surgery, and at day 2 (before euthanasia).	48 hours post-surgery.	-**Thoracic**: 4 dogs, 1 sample / dog.<br>- **Lumbar**: 4 dogs, 1 sample / dog.
Endo-vascular repair (EVR)	3	**Permanent** stenting of the descending aorta.	MRI + CTA before surgery, at day 2, and at day 9 (before euthanasia).	9 days post-surgery.	- **Thoracic**: 3 samples from dog#1 and dog#2, and 4 samples from dog #3.<br>- **Lumbar**: 3 samples from dog#1 and dog#2, and 4 samples from dog#3.

Endovascular surgical procedure

Dog#1: We introduced the 20-F Cook sheath 26 x 21 x 15 cm long C-tag gore device and deployed it to cover the origin of the left subclavian artery with its proximal edge overlapping the distal part of the origin of the innominate artery. That proved to be difficult due to the extreme angulation of the dog's aortic arch and the device was deployed distal to the left subclavian artery origin and was not able to cover it. We then introduced another device of the same size to cover the left subclavian origin that was successful but we found it to be very risky to perform routinely in future dogs. We then changed the view to lateral view and performed another angiogram to identify the origin of the celiac and superior mesenteric arteries. The third device 21 x 10 was deployed overlapping the previous one and the distal end was just proximal to the origin of the celiac artery. We did have difficulty retrieving the deployment system of the C-tag devices through the sheath where the olive of the device was stuck on the tip of the sheath. That is the reason we used Dry-seal Gore sheath in the next two dogs. At this point the wire and sheath was removed. The distal abdominal aorta and both external iliac vessels were clamped and the arteriotomy closed using running 4-0 prolene stitch in a transverse manner.

Dog#2: Based on our experience with the first dog, we decided to perform few changes to the technique. We decided to use Dry-seal Gore sheath for the reasons mentioned above. We also decided to use three 21 x 10 cm C-tag devices as the devices used in the first dog were thought to be too large for the dog's aorta. We also decided to plug the left subclavian artery using 12-mm Amplatzer-II device as covering the left subclavian artery with the endograft device was deemed difficult due to the acute angulation of the dog's aorta and the possibility of the device jumping forward during deployment to cover the innominate artery that would be fatal to the dog. We also decided to cover the proximal lumbar arteries (except the 5th pair, where the artery of Adamkiewicz usually merges [19]) using 10-mm Viabhan devices as they were no accessible to ligate surgically through the abdominal wall incision.

After placement of the stiff wire in the ascending aorta, we placed the Glidewire in a coaxial manner up to the aortic arch followed by a 55-cm 6-F cook sheath into the arch. Then using the wire and Bernstein catheter, we cannulated the left subclavian artery under flourscopic guidance. We then pushed the sheath into the left subclavian artery and performed left subclavian angiogram to identify the origin of the left vertebral artery. We then plugged the origin of the left subclavian artery using the fore-mentioned plug with its distal end proximal to the left vertebral artery. The 6-F sheath was then removed and we deployed three overlapping 21 x 10 C-tag devices with the first one proximal end just distal to the Amplatzer-II plug and the third one distal end just proximal to the celiac artery origin. At this point an abdominal aortogram was performed in the AP position to identify the origin of the bilateral renal arteries and deployed two 10 x 5 cm long Viabhan devices with the proximal edge just distal to the renal arteries and the distal end of the second one at the arteriotomy site. The sheath and wire were then removed and the arteriotomy closed in the same fashion as dog#1.

Dog#3: Based on our experience of the second dog, we decided to perform the procedure in the same fashion with one exception that is to make sure there is overlap between the two abdominal aortic Viabhan stents, as in the second dog, they did not overlap in order to leave the 5th lumbar segmental artery open. In dog#3, we deployed the distal Viabhan stent to overlap with the proximal one considering that we already ligated the distal 2 pairs of lumbar vessels surgically. Otherwise, the dog#3 was performed in the same way as dog#2.

Abdominal wall closure

After the endovascular portion was done, the vessel loops were removed, the Balfour retractor removed and the peritoneum allowed draping back in position after securing hemostasis. The muscular layer was closed using 2-0 Vicryl running stitches in two layers. Skin was then closed using running simple 2-0 sild stitches and a light dressing was applied. Bilateral femoral pulses were palpable at the end of the cases of all dogs.

Perfusion

The Perfusion portion of the lab was conducted using a Medtronic Bio-Console 560 pump and a Medtronic Affinity CP centrifugal pump. Twelve liters PBS solution was placed in a 19 liter graduated plastic container with ice. Four feet of 3/8" tubing was placed on the inflow port of the centrifugal pump. The tubing was connected to an 18" long plastic tube that was inserted through the cap of the PBS solution container so that the tip rested in the bottom corner of the

container. Eight feet of 3/8” tubing was placed on the outflow port of the centrifugal pump. This tubing was connected to an 18 Fr Redo EOPA Arterial cannula. The circuit was primed by tipping the PBS solution container on its side and allowing the solution to gravity feed through the circuit. The PBS container was then returned to an upright position and the cap was loosened. The circuit was then clamped until the connection to the cannula was complete and we were ready to initiate flow. Upon cannulation of the left ventricular apex flow was initiated slowly and increased until 1.5 – 2.0 lpm of flow was achieved. After approximately 30 seconds of flow the right atrial appendage was excised. Blood was allowed to freely exit the right atrial appendage and flow was continued until 4 liters of fluid was pumped through the canine subject. If the fluid exiting the appendage was not clear after 4 liters, flow was continued until such time that it was.

Supplementary Table 2. Anesthetics and analgesics agents administered before, during and after surgery.

Agent Name	Vehicle	Dose	Route	Volume	Frequency
Acepromazine	N/A	0.1-0.2 mg/kg	Intramuscular, subcutaneous	<5 ml	Pre-anesthetic medication
Bupernorphine	N/A	0.01-0.03 mg/kg	Intramuscular, intravenous, subcutaneous	<5ml	As needed during or after surgery
Bupivacaine	N/A	0.25%, 1-2mg/kg	Wound infilteration	5-15ml	At the end of each surgery
Carprofen	Tablet	2.2-4.4 mg/kg	oral, subcutaneous	<5ml	Every 12 hours postoperative
Cefazolin	N/A	20 mg/kg	Intravenous	<5 ml	30 minutes before skin incision and every 2 hours during surgery
Cetacaine	Cotton swab	200 mg	Topical	1-2 ml	Once after surgery
Fentanyl	Patch	50-100 ug/hr	Transdermal	N/A	Added 1 day before surgery, left for 72 hours.
Fentanyl	N/A	50 ug/hr	Intravenous	according to duration of surgery	During surgery
Heparin	N/A	2000 IU	Intravenous	<5 ml	Once before arotic clamping during second surgery of left thoracotomy
Isoflurane	Oxygen	0.5-5%	Inhalation	N/A	During surgery
Lidocaine 1%	N/A	1-2 mg/kg	Wound infiltration	5-15 ml	At the end of each surgery
Meloxicam	N/A	0.1-0.2 mg/kg	oral, subcutaneous	<5 ml	Every 24 hours postoperative
Propofol	N/A	2-6 mg/kg	Intravenous	<10 ml	On induction of anthesia for each procedure
Tramadol	Tablet	1-4 mg/kg	Oral	<5 ml	Every 6-12 hours

Metabolon Platform [18]

Sample Accessioning. Following receipt, samples were inventoried and immediately stored at -80°C. Each sample received was accessioned into the Metabolon LIMS system and was assigned by the LIMS a unique identifier that was associated with the original source identifier only. This identifier was used to track all sample handling, tasks, results, etc. The samples (and all derived aliquots) were tracked by the LIMS system. All portions of any sample were automatically assigned their own unique identifiers by the LIMS when a new task was created; the relationship of these samples was also tracked. All samples were maintained at -80°C until processed.

Sample Preparation. Samples were prepared using the automated MicroLab STAR® system from Hamilton Company. Several recovery standards were added prior to the first step in the extraction process for QC purposes. To remove protein, dissociate small molecules bound to protein or trapped in the precipitated protein matrix, and to recover chemically diverse metabolites, proteins were precipitated with methanol under vigorous shaking for 2 min (Glen Mills GenoGrinder 2000) followed by centrifugation. The resulting extract was divided into five fractions: two for analysis by two separate reverse phase (RP)/UPLC-MS/MS methods with positive ion mode electrospray ionization (ESI), one for analysis by RP/UPLC-MS/MS with negative ion mode ESI, one for analysis by HILIC/UPLC-MS/MS with negative ion mode ESI, and one sample was reserved for backup. Samples were placed briefly on a TurboVap® (Zymark) to remove the organic solvent. The sample extracts were stored overnight under nitrogen before preparation for analysis.

Quality controls (QCs). Several types of controls were analyzed in concert with the experimental samples: a pooled matrix sample generated by taking a small volume of each experimental sample (or alternatively, use of a pool of well-characterized human plasma) served as a technical replicate throughout the data set; extracted water samples served as process blanks; and a cocktail of QC standards that were carefully chosen not to interfere with the measurement of endogenous compounds were spiked into every analyzed sample, allowed instrument performance monitoring and aided chromatographic alignment. Supplementary Tables 3 and 4 describe these QC samples and standards. Instrument variability was determined by calculating the median relative standard deviation (RSD) for the standards that were added to each sample prior to injection into the mass spectrometers. Overall process variability was determined by calculating the median RSD for all endogenous metabolites (i.e., non-instrument standards) present in 100% of the pooled matrix samples. Experimental samples were randomized across the platform run with QC samples spaced evenly among the injections. For that purpose, a small aliquot of each client sample is pooled to create a CMTRX technical replicate sample, which is then injected periodically throughout the platform run. Variability among consistently detected biochemicals can be used to calculate an estimate of overall process and platform variability.

Ultrahigh Performance Liquid Chromatography-Tandem Mass Spectroscopy (UPLC-MS/MS). All methods utilized a Waters ACQUITY ultra-performance liquid chromatography (UPLC) and a Thermo Scientific Q-Exactive high resolution/accurate mass spectrometer interfaced with a heated electrospray ionization (HESI-II) source and Orbitrap mass analyzer

operated at 35,000 mass resolution. The sample extract was dried then reconstituted in solvents compatible to each of the four methods. Each reconstitution solvent contained a series of standards at fixed concentrations to ensure injection and chromatographic consistency. One aliquot was analyzed using acidic positive ion conditions, chromatographically optimized for more hydrophilic compounds. In this method, the extract was gradient eluted from a C18 column (Waters UPLC BEH C18-2.1x100 mm, 1.7 µm) using water and methanol, containing 0.05% perfluoropentanoic acid (PFPA) and 0.1% formic acid (FA). Another aliquot was also analyzed using acidic positive ion conditions, however it was chromatographically optimized for more hydrophobic compounds. In this method, the extract was gradient eluted from the same afore mentioned C18 column using methanol, acetonitrile, water, 0.05% PFPA and 0.01% FA and was operated at an overall higher organic content. Another aliquot was analyzed using basic negative ion optimized conditions using a separate dedicated C18 column. The basic extracts were gradient eluted from the column using methanol and water, however with 6.5mM Ammonium Bicarbonate at pH 8. The fourth aliquot was analyzed via negative ionization following elution from a HILIC column (Waters UPLC BEH Amide 2.1x150 mm, 1.7 µm) using a gradient consisting of water and acetonitrile with 10mM Ammonium Formate, pH 10.8. The MS analysis alternated between MS and data-dependent MS^n scans using dynamic exclusion. The scan range varied slighted between methods but covered 70-1000 m/z. Raw data files are archived and extracted as described below.

Bioinformatics. The informatics system consisted of four major components, the Laboratory Information Management System (LIMS), the data extraction and peak-identification software, data processing tools for QC and compound identification, and a collection of information interpretation and visualization tools for use by data analysts. The hardware and software foundations for these informatics components were the LAN backbone, and a database server running Oracle 10.2.0.1 Enterprise Edition.

LIMS. The purpose of the Metabolon LIMS system was to enable fully auditable laboratory automation through a secure, easy to use, and highly specialized system. The scope of the Metabolon LIMS system encompasses sample accessioning, sample preparation and instrumental analysis and reporting and advanced data analysis. All of the subsequent software systems are grounded in the LIMS data structures. It has been modified to leverage and interface with the in-house information extraction and data visualization systems, as well as third party instrumentation and data analysis software.

Data Extraction and Compound Identification. Raw data was extracted, peak-identified and QC processed using Metabolon's hardware and software. These systems are built on a web-service platform utilizing Microsoft's .NET technologies, which run on high-performance application servers and fiber-channel storage arrays in clusters to provide active failover and load-balancing. Compounds were identified by comparison to library entries of purified standards or recurrent unknown entities. Metabolon maintains a library based on authenticated standards that contains the retention time/index (RI), mass to charge ratio (*m/z)*, and chromatographic data (including MS/MS spectral data) on all molecules present in the library. Furthermore, biochemical identifications are based on three criteria: retention index within a narrow RI window of the proposed identification, accurate mass match to the library +/- 10 ppm, and the MS/MS forward

and reverse scores between the experimental data and authentic standards. The MS/MS scores are based on a comparison of the ions present in the experimental spectrum to the ions present in the library spectrum. While there may be similarities between these molecules based on one of these factors, the use of all three data points can be utilized to distinguish and differentiate biochemicals. More than 3300 commercially available purified standard compounds have been acquired and registered into LIMS for analysis on all platforms for determination of their analytical characteristics. Additional mass spectral entries have been created for structurally unnamed biochemicals, which have been identified by virtue of their recurrent nature (both chromatographic and mass spectral). These compounds have the potential to be identified by future acquisition of a matching purified standard or by classical structural analysis.

Curation. A variety of curation procedures were carried out to ensure that a high quality data set was made available for statistical analysis and data interpretation. The QC and curation processes were designed to ensure accurate and consistent identification of true chemical entities, and to remove those representing system artifacts, mis-assignments, and background noise. Metabolon data analysts use proprietary visualization and interpretation software to confirm the consistency of peak identification among the various samples. Library matches for each compound were checked for each sample and corrected if necessary.

Supplementary Table 3. Description of Metabolon QC samples

Type	Description	Purpose
MTRX	Large pool of human plasma maintained by Metabolon that has been characterized extensively.	Assure that all aspects of the Metabolon process are operating within specifications.
CMTRX	Pool created by taking a small aliquot from every customer sample.	Assess the effect of a non-plasma matrix on the Metabolon process and distinguish biological variability from process variability.
PRCS	Aliquot of ultra-pure water.	Process Blank used to assess the contribution to compound signals from the process.
SOLV	Aliquot of solvents used in extraction.	Solvent Blank used to segregate contamination sources in the extraction.

Supplementary Table 4. Metabolon QC standards

Type	Description	Purpose
RS	Recovery Standard	Assess variability and verify performance of extraction and instrumentation.
IS	Internal Standard	Assess variability and performance of instrument.
